# The dual function of microglial polarization and its treatment targets in ischemic stroke

**DOI:** 10.3389/fneur.2022.921705

**Published:** 2022-09-23

**Authors:** Yong Mo, Weilin Xu, Kaijing Fu, Hainan Chen, Jing Wen, Qianrong Huang, Fangzhou Guo, Ligen Mo, Jun Yan

**Affiliations:** ^1^Department of Neurosurgery, Guangxi Medical University Cancer Hospital, Nanning, China; ^2^Department of Neurosurgery, Second Affiliated Hospital, School of Medicine, Zhejiang University, Hangzhou, China; ^3^Department of Rheumatism, First Affiliated Hospital of Guangxi Medical University, Nanning, China

**Keywords:** ischemic stroke, treatment target, microglia, polarization, dual function

## Abstract

Stroke is the leading cause of disability and death worldwide, with ischemic stroke occurring in ~5% of the global population every year. Recently, many studies have been conducted on the inflammatory response after stroke. Microglial/macrophage polarization has a dual function and is critical to the pathology of ischemic stroke. Microglial/macrophage activation is important in reducing neuronal apoptosis, enhancing neurogenesis, and promoting functional recovery after ischemic stroke. In this review, we investigate the physiological characteristics and functions of microglia in the brain, the activation and phenotypic polarization of microglia and macrophages after stroke, the signaling mechanisms of polarization states, and the contribution of microglia to brain pathology and repair. We summarize recent advances in stroke-related microglia research, highlighting breakthroughs in therapeutic strategies for microglial responses after stroke, thereby providing new ideas for the treatment of ischemic stroke.

## Introduction

Stroke is the leading cause of mortality worldwide (1, 2) with poor curative effect, high lethality, and poor prognosis. Among all types of stroke, ischemic stroke caused by the occlusion of blood vessels represents the majority (3). Previous research has indicated that brain injury is caused not only by the hematoma mass effect and potential hematoma expansion (which are the main causes of primary brain injury) but also by secondary brain injury (SBI) (4). Cerebral ischemia can lead to a series of pathological processes including excitatory toxicity, calcium overload, oxygen free radical damage, inflammatory responses, necrosis/apoptosis, and blood–brain barrier (BBB) destruction, which ultimately lead to irreparable neuronal damage (5). It is now proposed that injury after stroke is a complex pathophysiological process involving several genes and signaling pathways. The BBB is important, and its permeability appears to follow a heterogeneous pattern of different stroke stages associated with different biological substrates. In the hyperacute phase, sudden hypoxia damages the BBB, leading to cytotoxic edema and increased permeability; in the acute phase, neuroinflammatory responses exacerbate BBB damage, leading to higher permeability and subsequent risk, which can be stimulated by reperfusion therapy; and in the subacute phase (1–3 weeks), repair mechanisms, particularly neovascularization, occur. BBB leakage occurs in immature vessels, but this permeability is associated with improved clinical recovery. In the chronic phase (>6 weeks), an increase in the BBB restoration factor causes the barrier to begin to reduce its permeability (6). Manipulation of microglial polarization is a potential treatment strategy for patients with ischemic stroke, but small- and medium-sized glial cells in the potential molecular mechanisms of the polarization in ischemic stroke are still controversial. Despite the simplicity of the experiment, more work and clinical trials are needed to fully understand the mechanisms of microglial polarization (7). Evaluating the best time to intervene with microglia and monocyte/macrophage therapeutic strategies against ischemic stroke, as well as determining how to stimulate cells and to polarize their states, as well as the role of microRNAs (miRNA) and transplanted stem cells in mediating microglial activation and polarization during cerebral ischemia, are all important topics for future research (8, 9). Targeting specific miRNAs may provide major restorative therapy, and microglia-based therapy for ischemic stroke may become a future research area.

Recent studies have shown that there are still no effective therapeutic targets to improve the neurological function of patients after stroke, and potential treatment methods for SBI remain a hot point of research. Currently, an effective treatment for ischemic stroke is mainly intravenous thrombolysis and mechanical thrombectomy. However, these treatment options are limited by the recommended treatment window (10, 11). In addition, a series of reperfusion injuries caused by inflammation and oxidative stress may occur after ischemia-reperfusion (12); oxidative stress can induce inflammation (13, 14). There is increasing evidence that, during cerebral infarction, persistent neuroinflammation damages neurons and the BBB, leading to tissue destruction and impaired function (15–17). Neuroinflammation plays a crucial role in ischemic stroke-induced brain injury and affects disease prognosis. Future research will focus on controlling stroke-induced inflammation by targeted drugs and will be challenging.

**Figure 1:**
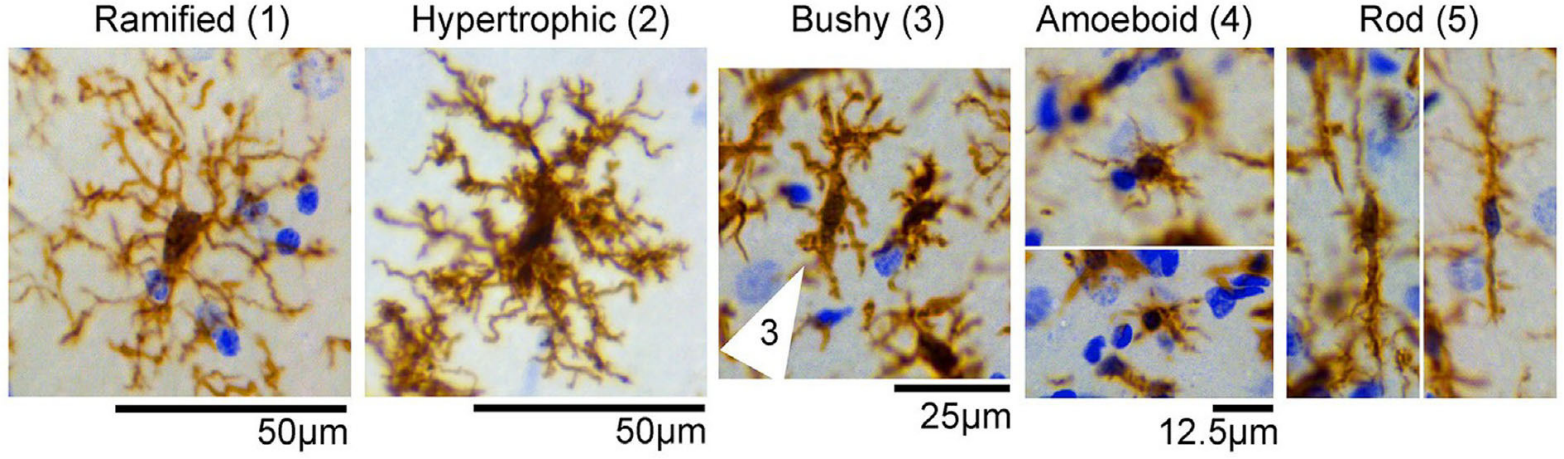
Representative images of microglial/macrophage cells (brown) with different morphologicalphenotypes observed in the control and status epilepticus (SE) groups, including (1) ramified, (2) hypertrophic, (3) bushy (cell indicated by arrows), (4) amoeboid, and (5) rod-shaped. Images were taken from the hippocampus of control or SE animals. Nissl-stained nuclei are indicated in blue [the figure is reproduced (19)].

Microglia are the permanent substantial macrophages in the central nervous system (CNS), and activated microglia typically behave “amoeba-like,” primed for action (18). Several findings showed that almost five different types of microglia morphology were identified in control and experimental status epilepticus (SE) tissues, and were categorized as follows: (1) ramified; (2) hypertrophic; (3) bushy; (4) amoeboid; and (5) rod-shaped (19) (Figure 1). Microglial polarization plays a major role in promoting brain injury and nerve recovery (20). As the main source of inflammatory cells in ischemic brain injury, microglia play a key role in the inflammatory response after stroke (21). After stroke, microglia are polarized to the classical pro-inflammatory type (M1-like) or the alternative protective type (M2-like) under optimal conditions (8). Classical M1-like microglia are related to the induction of pro-inflammatory molecules, while other M2-like microglial activations are related to neuroprotection (22). In this review, advances in microglia and ischemic stroke, including the dual functions of phenotypic polarization of microglia/macrophages and polarization-related signaling pathways, have been studied. Future ischemic stroke treatments may target microglial polarization in the future.

## Origin and function of microglial cells

Derived from primitive yolk sac progenitor cells, microglia are a type of fixed macrophages (9). The number of microglia showed a steady increase in the first 2 weeks after birth, and gradually decreased to 50% of the level at birth between 3 to 6 weeks later, after which the density gradually stabilized. A decrease in the rate of proliferation accompanied by an increase in apoptosis results in a decrease in the overall number of microglia, and mature microglia maintain their numbers in the CNS by self-renewal (23, 24). In the CNS, microglial cells in the brain of healthy adults are renewed to maintain their number and local expansion (25). In the physiological state, microglial cells present a typical branch-like state of small cell body and long branches and are referred to as “resting microglial cells.” The protrusions have high mobility and can carry out extensive and continuous monitoring of the surrounding environment. In the pathological state, microglial cells are changed from the resting state to the active state. Polarization refers to the fact that microglia are affected by exogenous substances to achieve a specific phenotype, and there are one or more molecular markers and significant changes in molecular distribution (26). M1-like and M2-like microglia are essential in tissue damage and repair, respectively. Polarization of M1-like and M2-like microglia is also considered a functional manifestation of CNS disease, which is specifically manifested in the release of CNS disease-related inflammatory factors and the role of neuroinflammatory responses (27–29).

## Microglial polarization

### Polarization of the M1-like phenotype

M1-like microglia can secrete a variety of pro-inflammatory factors and chemokines, which can cause a neuroinflammatory response and induce neuronal apoptosis (30). As for the classic excitation type, it is mainly induced by interferon-γ (INF-γ), lipopolysaccharides (LPS), and tumor necrosis factor-alpha (TNF-α), which is characterized by the production of several pro-inflammatory cytokines, such as interleukin-1β (IL-1β), IL-6, stromal cell-derived factor-1 (SDF-1/CXCL12), IL-1β, IL-12, and IL-23, and can be detected using cell surface markers such as cluster of differentiation 16 (CD16), CD32, major histocompatibility complex class II (MHCII), CD86, TNF-α, inducible nitric oxide synthase (iNOS), etc. During ischemia/hypoxia, nuclear factor-κB (NF-κB) is activated in microglia and transferred from the cytoplasm to the nucleus. This activates the release of pro-inflammatory cytokines that lead to SBI (31–33), such as IL-1β, IL-6, TNF-α, and iNOS. In addition, TNF-α secreted by M1-like microglia was identified to increase endothelial necrosis and BBB leakage after ischemic stroke in middle cerebral artery occlusion (MCAO) model mice. This further promotes neuroinflammation and cerebral edema, leading to poor outcomes (34, 35). Classically activated microglia can perform pro-inflammation, phagocytosis, cytotoxicity, present antigens, and kill intracellular pathogens to maintain the homeostasis of the microenvironment (36, 37). Notably, the M1-like phenotype of microglia is usually associated with protection during the early acute stages of infection, but it can also be detrimental to the host in case of its persistence for a longer time. Changes in the expression of corresponding proteins also follow a similar course node (38–40). If homeostasis is destroyed or stimulation persists, inflammatory cascades can be induced, resulting in the massive release of inflammatory factors and neurotoxic substances, aggravating the inflammatory response, and inducing neuronal death (36, 37) (Table 1).

**Table 1:** Characteristics of M1 microglia and M2 microglia in ischemic stroke.

**Phenotype**	**Markers**	**Mechanism**	**Effects**
M1	CD16, CD32, CD86, IL-1β, IL-6, TNF-α, iNOS, MHCII, et al.	NF-κB is activated in microglia and transferred from cytoplasm to nucleus, activating the release of pro-inflammatory cytokines such as IL-1β, IL-6, and TNF-α. TNF-α increases endothelial necrosis and BBB leakage	Proinflammatory, phagocytosis, cytotoxicity, present antigens, and kill intracellular pathogens
M2	CD206, Arg-1, TGF-β, CD163, IGF-1, IL-10, et al.	PParγ was activated in microglia and moved from nucleus to cytoplasm, resulting in the release of anti-inflammatory cytokines from M2. The up-regulation of TGF-α expression promoted the proliferation and neuronal differentiation of nerve stem/progenitor cells in the inferior ipsilateral ventricle	Anti-inflammatory, nerve repair, and tissue remodeling

CD, Cluster of Differentiation; IL, interleukin; TNF-α, Tumor necrosis factor-α; iNOS, Inducible nitric oxide synthase; MHCII, Major histocompatibility complex class II; Arg-1, Arginase-1; TGF-β, Transforming growth factor-β; IGF-1, Insulin growth factor 1.

### Polarization of the M2-like phenotype

**Figure 2:**
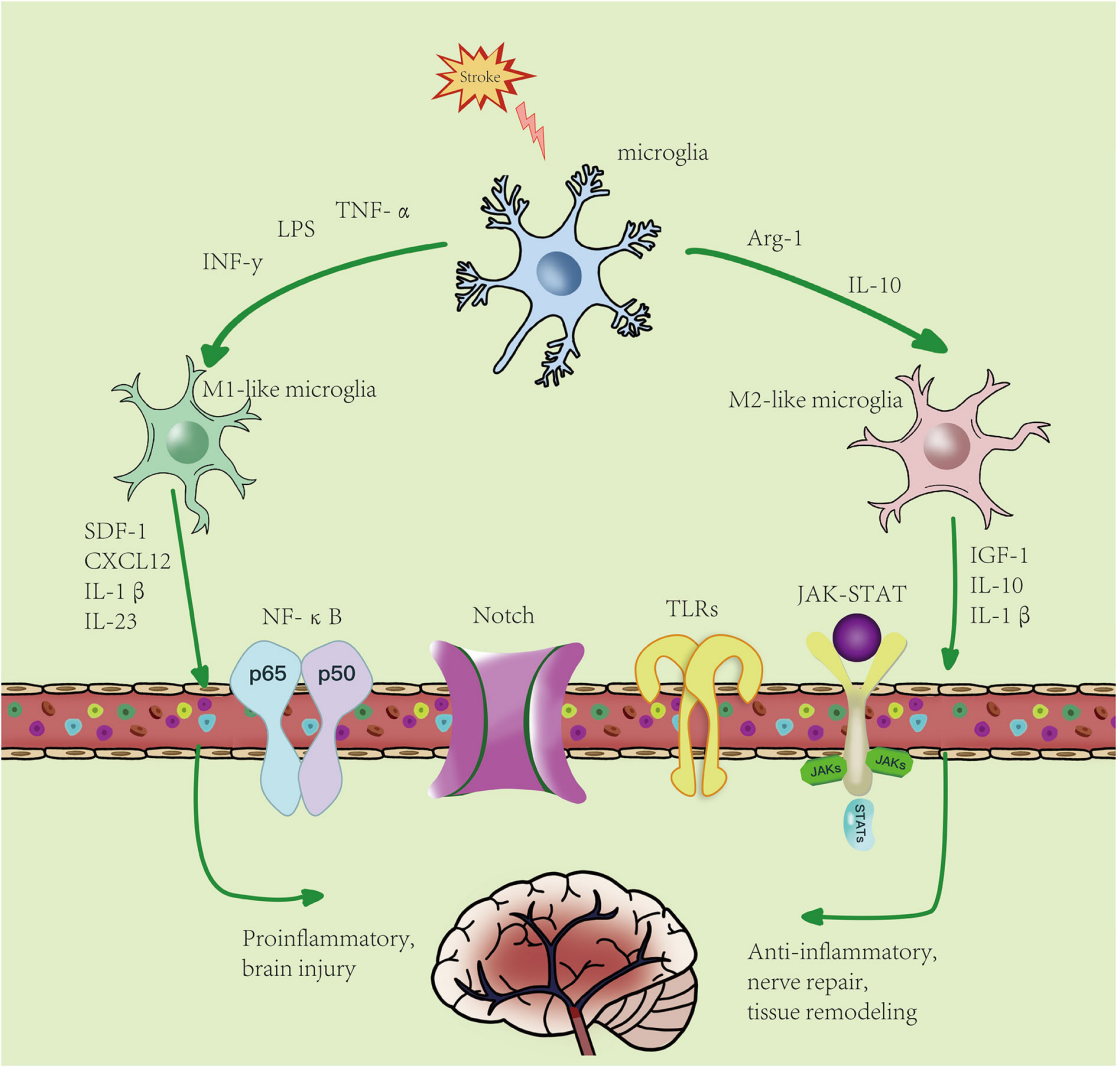
Microglial polarization after stroke: microglial activation is divided into two phenotypes: M1-like and M2-like microglia. M1 microglia can be induced by lipopolysaccharides (LPS), interferon-γ (IFN-γ), etc., resulting in an increase in pro-inflammatory factors. M2-like microglia can be induced by IL-4, IL-13, etc., resulting in an increase in anti-inflammatory factors. Activated microglia pass through NF-κB, JAK-STAT, Notch, TLRs, and other signaling pathways. M1-like type promotes the inflammatory response and kills intracellular substances, while M2-like type plays an anti-inflammatory, neuroprotective, and repairing role in tissues.

According to the unique functions of microglia/macrophages and their gene expression profiles, there are four different types of polarized activation states of M2-like activated phenotype: M2a microglia, M2b microglia, M2c microglia, and M2d microglia (41). There are many markers in M2-like microglia, such as CD206, increased arginase 1 (Arg-1), TGF-β, insulin growth factor 1 (IGF-1), IL-10, and others, and they secrete anti-inflammatory cytokines and neurotrophic factors, such as IL-10β, brain- and glial cell-derived neurotrophic factors, and Arg-1, the expression of factors such as IGF-1, thereby inhibiting inflammation (42), involved in tissue repair, cell debris removal, tissue remodeling, the provision of nutritional factors, and the maintenance of tissue dynamics after infection or injury (43, 44). In general, M2-like microglia can be identified by CD206 and Arg1, IGF-1, among other markers (45). M2a is produced by IL-4 and IL-13 stimulation and inhibits NF-κB signal transduction and the anti-inflammatory phenotype of activated B cells. Moreover, M2a is involved in parasite immunity, T helper 2 cell recruitment, tissue repair, and growth stimulation. M2b is produced by stimulating immune complexes and LPSs to secrete anti-inflammatory cytokines (such as IL-10, MHC II, and co-stimulatory CD86). This subset exhibits both pro- and anti-inflammatory features and is associated with adaptive immunity. M2c is activated by IL-10 and TGF- β. Arg-1, CD163, and CD206 are the markers of M2c cells, which mainly function in scavenging cell debris during the repair process and is related to immunosuppression and tissue remodeling (46–48). M2c is different from the M2-like subtypes described earlier and is produced by activating the activation state of adenosine A2a receptor (A2aR) in M1 pro-inflammatory cells. M2d is different from M2-like subtypes described earlier and is produced by activating the activation state of A2aR in M1 pro-inflammatory cells (49). Peroxisome proliferator-activated receptor γ (PPARγ), a transcription factor with anti-inflammatory properties, is activated in microglia and translocated from the nucleus to the cytoplasm under ischemia/hypoxia conditions (50). This leads to the activation of M2-like microglia, which release anti-inflammatory cytokines and improve stroke outcomes. In addition, Choi et al. (51) demonstrated that M2-like microglia promoted the proliferation and neuronal differentiation of nerve stem/progenitor cells in the ipsilateral subventricular region after ischemic stroke by upregulating the TGF-α expression level, which may provide an effective treatment for neurogenesis (Figure 2).

## The transition between M1 and M2

A shift from M2 to M1 has been observed in models of traumatic brain injury and ischemic stroke; however, it re mains to be determined whether this transformation is caused by phenotypic transformation of individual microglia or by the migration and infiltration of M2-like microglia (52). Studies have shown that in ischemic stroke, activated microglia express M2-like microglia markers in the acute phase. However, within ~1 week, a gradual transition to M1-like phenotypes occurs and persists for several weeks after injury, this phenotypic transition may be due to the recruitment of M1-like microglia to the injury site and the transformation of locally activated microglia from M2 to M1 cells (53). Therefore, selective neuro-immunomodulatory therapies, which largely focus on suppressing M1-like phenotypes and shifting microglia from the M1-like phenotype to the M2-like phenotype, have been proposed as neuroprotective strategies for stroke (54). Furthermore, an experiment showed that the silencing of NF-κB p65 downregulated the expression of M1-like biomarkers and promoted the expression of M2-like biomarkers in the *in vitro* and *in vivo* model of cerebral ischemia (55).

Recently, a mouse model of transient focal cerebral ischemia has been used to study the temporal dynamics of microglial/macrophage polarization after stroke. Research results suggest that microglia/macrophages respond dynamically to ischemic injury, experiencing an early “healthy” M2-like phenotype, followed by a transition to a “sick” M1-like phenotype (56). *In vivo* temporal distribution of increased iNOS and chitinase-like protein 3 (Chil3; Ym 1) promoter activity in the mouse brain (57). The relatively low iNOS signal in healthy brain increased ~3-fold within 3 days of stroke induction, whereas Ym1 signal reached a maximum at 11–13 days after stroke induction and then declined over the following week. Hu et al. found an early increase of iNOS messenger RNA (mRNA) levels as well as of other pro-inflammatory markers, such as CD16, CD32, CD86, and CD11b beginning at 3 dps and continuing up to 14 dps, with the exception of CD86. They also reported that chitinase-3-like protein 3 (Ym1/2) mRNA levels peaked on day 3 and then declined up to 14 dps (56).

## Mechanism of microglial polarization in ischemic stroke

### Signaling pathways that regulate microglial polarization

#### NF-κB signaling pathways

Studies have shown that in mammals there are all five members of the NF-κB family including NF-κB1 (p105/p50), RelA (P65), and NF-κB2 (p100/p52), which are composed of homo- and heterodimers. In contrast to the c-NF-κB dimers, the p65/p50 heterodimer RelB is the most classical form of existence. It exists in most cell types and plays the most important role as an effective transcription factor. The activation of NF-κB is required for transcriptional induction of many pro-inflammatory mediators, such as IL-6, iNOS, intercellular adhesion molecule 1 (ICAM1), matrix metalloproteinase 9 (MMP-9), and cyclooxygenase-2 (COX-2), which are involved in innate immunity. A previous study demonstrated that the NF-κB signaling pathway was overactivated in microglia after ischemic stroke. Therefore, the activation of NF-κB was responsible for the polarization of M1 and M2 in microglia (58, 59). In p50 KO mice, NF-κB activation exacerbated ischemic neuronal damage, especially in microglia. NF-κB p65 and p50 form heterodimers to initiate pro-inflammatory responses, thereby enhancing M1-like activation and attenuating microglial M2-like responses (60). The activation of the NF-κB signaling pathway promotes the conversion of microglia to M1-like type, and effective inhibition of the activation of the signaling pathway is more conducive to the conversion of microglia to M2-like type (61). Studies have shown that the inhibition of the NF-κB signaling pathway or the expression of NF-κBp65 and IκBα or interference with the nuclear metastasis of NF-κB can inhibit the activation of microglia and the expression of the M1-like phenotype, reduce the expression of inflammatory factors such as IL-1β, IL-6, TNF-α in microglia, and have neuroprotective effects (62, 63). Therefore, the suppression of neuroinflammation and the amelioration of brain injury by inhibiting the expression and activity of NF-κB in microglia after ischemic stroke has become a breakthrough target for therapeutic strategies (Table 2).

**Table 2:** Overview of signaling pathways and their associated molecules.

**Signaling pathways**	**Composition structure**	**Signal molecules**	**Activation paths**	**Clinical effects**
**NF-κB**	NF-κB1, NF-κB2, NF-κB3, et al.	IL-6, iNOS, ICAM1, MMP-9, COX-2, et al.	Enhances M1 activation, attenuates M2 response	Inhibits inflammatory response and increases neuroprotection
**JAK – STAT**	JAKs and STAT1–6	INF-γ, LPS, IL-10, IL-4	Selectively activate M1 and M2	Reduce the release of inflammatory factors and accelerate the repair of damaged nerves
**Notch**	Four Notch receptors (Notch 1–4) and five Notch ligands (Delta type 1, 3, 4, sawtooth 1, 2)	unknow	Inhibit transition for M1–M2	Promote the release of inflammatory transmitters and aggravate nerve tissue damage
**TLRs**	a C-terminal TIR domain, a transmembrane region and an extracellular N-terminal	Pathogen-associated moleculars	Activation signaling pathways of NF-κB and MAPK	Increases pro-inflammatory factors and aggravates nerve damage

IL, interleukin; ICAM1, intercellular cell adhesion molecule-1; MMP-9, Matrix Metalloproteinase 9; COX-2, Cyclooxygenase-2; INF-γ, Interferon-γ; LPS, Lipopolysaccharide; MAPK, Mitogen-activated protein kinase.

#### Janus kinase/signal transducer and activator of transcription pathway

Signal transducer and activator of transcription (STAT) is phosphorylated by Janus kinase (JAK), dimerized, and then transported to the nucleus through the nuclear membrane to regulate the expression of related genes. This pathway is termed the JAK/STAT signaling pathway (64). STAT plays a key role in signal activation and transcription. The STAT family in the cytoplasm is a downstream target of JAKs, which is one of the most crucial cytokine-activated transcription factors in the process of immune response. It is composed of seven members, namely STAT1, STAT2, STAT3, STAT4, STAT5A, STAT5B, and STAT6 (65). STAT1, STAT3, and STAT6 members of the STAT family are involved in the polarization of microglia. IFN-γ can induce microglia to polarize toward M1-like type through the STAT1/STAT3 pathway, and release inflammatory factors such as iNOS/nitrous oxide (NO) at a higher level than normal cells (66). STAT1 responds to M1-like microglial polarization signals (INF-γ and LPS), while STAT3 and STAT6 are selectively activated by M2-like microglial polarization cytokines (IL-10, IL-4, etc.). Thus, the release of inflammatory factors can be reduced and the injured nerve repair can be accelerated (67). Considering the data related to JAK/STAT and autoimmune diseases, this method is extremely attractive to the pharmaceutical industry, which is also one of its goals.

#### Notch signaling pathway

The extracellular domain of Notch is composed of epidermal growth factors (EGFs) like repeats, the number of which varies among species and different Notch receptors. Two functional domains are present in the extracellular region, the ligand-binding domain (EGF 11–12), which mediates the interaction with ligands, and the Abruptex domain (EGF 24–29), whose function remains unclear. The extracellular region is followed by the negative regulatory region (NRR), which masks a cleavage site (S2) important for Notch activation, the heterodimerization domain (HD), and the transmembrane spanning region of the receptor (64, 68). The Notch signal pathway is an important signal transduction pathway that begins with the binding of the Notch receptor and ligand and then forms a transcriptional activation complex through interactions with transcription factors, which activates the target genes of the transcriptional suppressor family (e.g., HES, HEY, NERP, etc.) to play a transcriptional inhibitory role. In mammals, there are four Notch receptors (Notch 1–4) and five Notch ligands (Delta type 1, 3, 4, sawtooth 1, 2). Notch signaling can regulate the differentiation and development of cells, tissues, and associated cells. These cells include neurons, oligodendrocytes, astrocytes, and microglia. In the pathological state, the Notch pathway can promote the release of inflammatory transmitters and aggravate tissue damage by activating microglia and inhibiting the transformation of M1-like to M2-like microglia (69). It was confirmed in experiments of BV2 microglia-related cells that the release of inflammatory mediators from M1-like microglia decreased and converted to M2-like microglia after the use of a Notch signaling antagonist. Meanwhile, there is an increase in anti-inflammatory cytokines released by M2-like microglia. This confirms the involvement of the Notch pathway in the inflammatory response following microglial activation (70).

#### Toll-like receptor signaling pathway

Toll-like receptors, named after the Toll proteins in *Drosophila melanogaster* (13), are a class of inherent immune recognition receptors that detects microbial pathogens associated with molecular patterns to induce an immune response (71). TLR is expressed on neurons in glial cells (microglia, astrocytes, and oligodendrocytes), the CNS, and the peripheral nervous system (PNS) (72). TLRs are type I transmembrane proteins composed of a C-terminal TIR domain, a transmembrane region, and an extracellular N-terminal. An extracellular N-terminal mainly recognizes extracellular pathogens and tissue damage signals. TLR4 in human microglia can recognize pathogen-associated molecular models and activate nonspecific immunity in ischemic brain injury via a myeloid differentiation factor pathway, and both NF-κB and mitogen-activated protein kinase (MAPK) signaling pathways are activated and participate in the inflammatory response. TLR4 receptors can repeatedly recognize different pathogen-related molecular patterns through extracellular leucine (73), and ultimately lead to the production of NF-κB and an increase in pro-inflammatory factors, and the secretion of serotonin may aggravate nerve damage (74).

### Regulatory mechanisms of microglial polarization

In addition to the influence of the abovementioned signaling pathways, there are also several regulatory mechanisms of microglial polarization: transcription factors, the regulation of gene expression, ion channels, and autophagy (75). Firstly, transcription factors, it was found that nuclear factor erythroid 2-related factor 2 (Nrf2) activation reduced the expression levels of reactive oxygen species (ROS), nucleotide-binding oligomerization domain- (NOD-) like receptor family Pyrin domain 3 (NLRP3), and IL-1 β in BV2 microglia, and played a protective role after ischemic stroke (12). In acute ischemic stroke, PPARγ is activated to directly reduce tissue damage by inhibiting the NF-κB pathway, reducing inflammation, and stimulating the Nrf2/ARE axis to reduce oxidative stress (76). IL-4 produced by neurons was determined to bind to IL-4 receptors expressed on microglia surfaces and activate M2-like microglia by modulating the PPARγ signaling pathway to reduce ischemic brain injury (77). Second, ion channel expression changes in response to voltage and pH gradients in the microenvironment, thereby inducing intracellular signal transduction. Currently, the two important ion channels Hv1 and Kv1.3 are closely related to microglial polarization. Studies have shown that Hvl can aggravate brain injury by increasing the expression levels of ROS and pro-inflammatory cytokines produced by M1-like microglia. However, it remains unclear whether Hv1 affects the polarization of M2-like microglia. The Kv1.3 inhibitor 5-(4-phenoxybutyl-psoralen) pSORalen (PAP-1) decreased the polarization of M1-like microglia and the expression level of pro-inflammatory cytokines. This also suggests that Kv1.3 may be one of the major mediators of the polarization of M1-like microglia (78, 79). Third, miRNA-155 and miRNA-124 in gene expression regulators are closely related to microglial polarization in ischemic stroke. The expression levels of miRNA-155 were significantly increased in LPS-activated microglia, which might target the inhibition of cytokine signaling to trigger M1-like microglia-mediated inflammation and aggravate brain injury (33). miRNA-124 induces neuroprotection and functional improvement by regulating M2-like microglial polarization in ischemic stroke (80). Finally, autophagy is a cellular metabolic pathway by which damaged organelles and misfolded proteins are degraded and recycled to maintain cellular homeostasis. Studies have shown that autophagy is activated in neurons, endothelial cells, microglia, and other brain cells in ischemic stroke and that interference with autophagy can aggravate brain injury. Studies have shown that autophagy may stimulate the transformation of microglia to the M1-like phenotype, thereby exacerbating cerebral ischemia. However, the role of autophagy in microglial polarization in ischemic stroke requires further investigation (81).

### Treatment targets of microglial polarization in ischemic stroke

Currently, there are numerous studies on stroke treatment, including extensive research on small molecules. For example, the small molecule miRNA-124 can regulate the activation state of microglia/macrophages, thereby improving stroke recovery (82). Chemokine-like factor 1 (CKLF1) is an important mediator that skews microglia/macrophages toward the M1-like phenotype in the early stage of cerebral ischemic injury, and targeting CKLF1 may also be a novel approach for IS treatment (83). Cytokine IL-4 may improve long-term neurological outcomes after stroke by inducing the M2-like phenotype in microglia/macrophages (84). In addition, the current treatment of SBI after stroke has become more promising. The inhibition of the inflammatory response promotes M2-like microglial polarization, reduces M1-like activation, and promotes the clearance of hematoma, thus playing a therapeutic role. The role of microglia-mediated inflammation in the undamaged CNS remains a hot spot of research. The development of multi-treatment targets is likely to become an important direction for the development of new therapeutic targets for ischemic stroke (Table 3).

**Table 3:** Therapeutic goals and related mechanisms of drugs in ischemic stroke.

**Drugs**	**Mechanisms**	**Polarization pathway**	**Therapeutic effects**	**Clinical aspects**
Minocycline	Inhibiting nuclear translocation of NF-KB, regulates STAT1/STAT6 pathway	Reduces the production of M1 and enhances the expression of M2	Inhibiting the activation and activation of microglia, the production of reactive oxygen species and cell apoptosis	Reducing the brain water content and brain edema, improve functional recovery in ischemic stroke
Metformin	Inhibits the inflammatory pathway mediated by brain NF-κB	M2	Reduces infarct volume and improves neurological deficits, promoting tissue repair	Chronic post-stroke therapy
rosiglitazone	Unknown	Promotes polarization of microglia toward the M2 phenotype	Educing oxidative stress, attenuating excitotoxicity	Improve white matter integrity after stroke, contributing to stroke long-term recovery
Dexmedetomidine	Unknown	Unknown	Diminish neuroinflammation in the mouse brain	Neuroprotective effect
Etifoxine	Unknown	Unknown	Reduce leukocyte infiltration, control the production of pro-inflammatory cells in microglia, improve the integrity of the blood-brain barrier	Reduce neurological deficits and infarct volume, limit brain inflammation, and provide protection against ischemia/reperfusion injury

Minocycline, an antibiotic of the tetracycline family, is known for its anti-inflammatory effects in neurological disorders, and has been reported to potentially improve functional recovery in ischemic stroke (85, 86). Minocycline can cross the BBB, accumulate in CNS cells, and inhibit microglia activation and proliferation, as well as MMP concentration and activity (85). Anti-inflammatory effects of minocycline have been demonstrated in neurological diseases in experimental models of ischemia, traumatic brain injury, and neuropathic pain as well as in Alzheimer's disease, Parkinson's disease, multiple sclerosis, Huntington's disease, amyotrophic lateral sclerosis, and several neurodegenerative diseases including spinal cord injury (87–93) (Table 4). Various experimental animal models and clinical trials have shown that minocycline can effectively cross the BBB, lead to the production of ROS and apoptosis by inhibiting the activation of microglia, and play a neuroprotective role against nervous system injury (94, 95). It has been reported that minocycline partially suppressed the production of inflammatory molecules (IL-6, TNF-A, and IL-1B) induced by LPS in peripheral monocytes by inhibiting nuclear translocation of NF-κB (96). In addition, experiments have shown that minocycline regulates M1/M2 microglial polarization through the STAT1/STAT6 pathway, reduces the production of M1-like polarization genes and enhances the expression of M2-like polarization genes by regulating STAT1 and STAT6 signaling, thus achieving the treatment of ischemia (97). Minocycline can effectively inhibit the diffusion of the neuroinflammatory cytokines IL-1β and NO, thereby reducing the brain water content and alleviating early brain edema and brain injury in the early stages of stroke by reducing the M1-like polarization of microglia (98).

**Table 4:** Summary of microglial polarization in neurological disorders.

**Neurological disorders**	**Markers**	**Mechanism**	**Effects of microglial polarization**
Alzheimer's disease (AD)	CD40, CD11c, CD33	Aβ clearance or Aβ clearance	neurodegeneration and cognitive impairment
Parkinson's disease (PD)	TNF-α, IL-6, CD36	May be similar to mechanism in AD	a double-edged sword
Multiple sclerosis (MS)	TGF-α	Microglia release proteases, pro-inflammatory cytokines, ROS, and RNS, and recruit reactive T lymphocytes	M1 microglia have enhanced antigen-presenting capacity, leading to demyelination and neurodegeneration. While M2 microglia protect oligodendrocytes and neurons from damage and improve disease severity
Huntington's disease (HD)	IL-6, TNFmRNA73	Microglia express higher HTT mRNA	Exacerbate neurodegeneration
Amyotrophic lateral sclerosis (ALS)	TGF-α	mSOD1 expression in microglia	Elimination of apoptotic cells, production of growth factors, maintenance of synapse structure and function are the main function of microglia

CD, Cluster of Differentiation; TNF-α, Tumor necrosis factor-α; IL, interleukin; TGF-α, Transforming growth factor-α; TNFmRNA73, Tumor necrosis factor mRNA73; Aβ, Amyloid-β; ROS, Reactive oxygen species; RNS, Reactive nitrogen species; HTT mRNA, Huntingtin mRNA; mSOD1, Mutant human superoxide dismutase1.

Metformin, a well-known AMP-activated protein kinase (AMPK) activator, can be used in chronic post-stroke therapy to promote functional recovery after experimental stroke. Experimental evidence suggests that post-stroke metformin treatment results in a long-term elevation of M2-like signature gene expression and the suppression of M1-like signature gene expression. Metformin enhances the M2-like polarized function of microglia/macrophages involved in tissue repair and is beneficial in ischemic stroke, thereby improving post-stroke brain function recovery. Therefore, promoting the functional phenotype of microglia tilted toward M2-like polarization *via* AMPK activation after stroke emerges as a novel therapeutic strategy for stroke (99). Animal experiments have also shown that, in chronic ischemic stroke, metformin pretreatment inhibits the inflammatory pathway mediated by brain NF-κB, which is accompanied by a reduction in pro-inflammatory cytokines, such as TNF-α, IL-1β, IL-6, and others. This significantly reduces infarct volume and improves neurological deficits while also promoting tissue repair (100).

Peroxisome proliferator-activated receptor, a ligand-activated transcription factor belonging to the nuclear receptor superfamily, has been shown to orchestrate the macrophage phenotype switch, thus leading to the inhibition of inflammation and tissue repair. Its agonist, rosiglitazone, promotes the polarization of microglia toward M2-like phenotype with a direct and indirect effect on the white matter. It may improve white matter integrity after stroke. In addition, it can reduce cerebral infarct size and edema in different animal models of stroke through the nuclear receptor PPAR-γ, thereby protecting neurons, reducing oxidative stress, attenuating excitotoxicity, and contributing to long-term recovery from stroke (44, 101). Unfortunately, the mechanism by which rosiglitazone improves stroke prognosis is still unknown and needs to be further explored.

Dexmedetomidine (DEX) is an α-adrenergic receptor agonist with different properties, including sedative, anxiolytic, antisympathetic, and analgesic, widely used as an adjuvant in the perioperative period (102). In a model of LPS-induced inflammation, many previous studies have reported that DEX can diminish neuroinflammation in the mouse brain and to modulate cytokine-associated changes in sickness behavior (103). In addition, it has been experimentally confirmed that in microglia, LPS induces a pro-inflammatory response through activation of the MAPK and NF-κB pathways (104). However, it remains to be further explored whether microglial polarization after stroke exerts neuroprotective effects through the abovementioned pathways, and the effect of clinically relevant concentrations of DEX on microglial M1/M2 polarization remains to be further investigated.

Etifoxine, a benzoxazine-based anti-anxiety compound, is an exogenous ligand of the 18-kDa translocator protein (TSPO) with high affinity (105). TSPO principally affects microglia (106). Experiments have confirmed that etifoxine can reduce brain damage and inflammation after stroke, reduce leukocyte infiltration, control the production of pro-inflammatory cells in microglia, improve BBB integrity, and reduce nerve cell death during hemorrhagic stroke. Thus, the function of repairing damaged nerves is achieved. In addition to finding reduced brain inflammation and altered microglial responses following etifoxine treatment, this still has been confirmed in mouse experiments. Together, these results demonstrate the therapeutic potential of etifoxine to reduce neurological deficits and infarct volume, limit brain inflammation, and provide protection against ischemia/reperfusion (I/R) injury. However, the mechanism of its effect needs further investigation (107).

In recent years, some traditional Chinese medicine formulations have also greatly improved post-stroke symptoms by promoting M2-like polarization. A novel resveratrol oligomer, named malibatol A, can reduce infarct size after MCAO in ischemic stroke (108) and increases M2-like microglial polarization markers such as CD206 and YM-1, producing anti-inflammatory protection. Its neuroprotective effect is largely associated with PPARγ-dependent activation of M2-like microglial polarization (44). The results of another similar study showed that the pharmacologically active component (hyperforin) of the medicinal plant Hypericum perforatum (St. John's wort) reduced infarct volume and induced microglia from M1-like to M2-like phenotype via the inhibition of IL-17A (109). Meisoindigo, a second-generation derivative of indierythroid (110), modulates microglial/macrophage polarization by inhibiting TLR4/NF-κB, reducing ischemic stroke-induced brain injury *in vivo* and *in vitro*. In addition, Meisoindigo has a neuroprotective effect in the ischemic brain. This protective effect is attributed to the inhibition of NOD-like receptor protein 3 (NLRP3) (111) inflammasome activation and the prevention of microglia/macrophages from the pro-inflammatory M1-like phenotype to the protective M2-like phenotype to relieve the inflammation in the brain (112, 113). If the mechanism of action of these drugs is understood accurately, ischemic stroke will be treated better.

In addition, recent studies have shown that inflammasome inhibitors have also been crucial treatment targets of microglial polarization in ischemic stroke, and NLRP3 inflammasomes have been proven to play a role in ischemic stroke. JLX001, a novel compound structurally similar to cycloviral flavonoid D (CVB-D), inhibits the expression of NLRP3 and proteins associated with the NLRP3 inflammasome axis *in vivo*, promoting a transition to a microglial M2 phenotype, suggesting that JLX001 is a promising treatment for ischemic stroke (114). Treatment with the LPR3 inflammasome inhibitor tranilast reduces the expression of M1 markers and pro-inflammatory cytokines, while stimulating the expression of M2-like microglia markers, thereby ameliorating ischemic stroke (115). Acute treatment with NLRP3-specific drugs, such as MCC950, reduces neuroinflammation in IS and improves neurological outcomes after stroke (116). These outcomes may also provide targeted therapeutic opportunities for stroke-related inflammation; however, research on the role of inflammasome inhibitors against ischemic stroke is still a long way off.

In one study, it was found that the body's circulating steroid, dehydroepiandrosterone (DHEA), can penetrate the BBB, and the inflammatory response of microglia is regulated by phosphorylation of tropomyosin-associated kinase A (TrkA) and subsequent activation of pathways involving protein kinase B 1/protein kinase B 2 (Akt1/Akt2) cAMP response element-binding proteins. The latter induces the expression of Histone 3 Lysine 27 (H3K27) demethylase Jumonji D3 (Jmjd3), which enhances the polarization of M2-like microglia and may contribute to phenotype conversion in microglia. Thus, the expression of inflammation-related genes and microglial polarization were controlled, thus providing a platform for future therapeutic interventions in neuroinflammatory pathology (117, 118). Recent studies on single-cell analysis suggest that microglia are spatially and developmentally heterogeneous, have time-specific and region-dependent subtypes (119), and exhibit distinct genetic characteristics associated with changes in the CNS microenvironment (120). Heterogeneous subsets of microglia may provide a new pathway for microglia to target neuroinflammation (121, 122).

As mentioned earlier, TLR2/4 on microglia are important regulators of inflammatory responses during cerebral I/R. TLR2 and TLR4 were found to be significantly elevated during reperfusion injury, which was associated with the degree of ischemic injury and inflammation (123). TLR can also interact with endogenous and exogenous molecules released during ischemia to increase tissue damage. In addition, TLR2 and TLR4 activate different downstream inflammatory signaling pathways. The relationship between neurosteroids and TLR after ischemic events may serve as a therapeutic target for stroke therapy (124). Meanwhile, inflammatory signaling of TLR2 in the ischemic brain requires the scavenger receptor CD36. It is possible to suppress inflammation by not having this receptor. These findings suggest that the TLR2-CD36 complex can act as a sensor for ischemia at the onset of death signals and is critical for inflammatory responses (72). Therefore, TLR2 inhibition may be considered as the future treatment for ischemic stroke. TLR2 and TLR4 signaling appears to be important in controlling pathogenic immune responses after stroke, and estrogen, progesterone, and vitamin D3 all regulate TLR2 and TLR4 signaling, making them therapeutic options for stroke treatment (72).

In addition, after stroke, the immune response induces inflammation, which is one of the main reasons for the progression of ischemic injury. Microglia are involved in the inflammation of the brain and have a bone marrow source (125). A focus of current research is the trigger receptor 2 (TREM2) expressed on myeloid cells. TREM2 is a cell-surface receptor, a unidirectional transmembrane receptor, belonging to the immunoglobulin-like receptor superfamily. In the CNS, it is mainly expressed on microglia (126). The activation of trigger receptors expressed on TREM2 stimulates microglial phagocytic activity and downregulates the expression of TNF-α and inducible iNOS (8). TREM2 overexpression has been shown to have the opposite effect, while TREM2 deficiency attenuates microglial phagocytic activity and exacerbates ischemic damage in experimental stroke (127). TREM2 overexpression significantly inhibits the inflammatory response and neuronal apoptosis in cerebral I/R injury (125). Docosahexaenoic acid (DHA) treatment enhances mesencephalic astrocyte-derived neurotrophic factor (MANF), reduces the expression of TREM2 and ischemic brain damage, activates neurogenesis, and promotes functional recovery after experimental ischemic stroke (128). These findings suggest that TREM2 is an attractive target for microglia regulation in the treatment of ischemic stroke, which may be a promising therapeutic strategy (129).

### Conclusion

After ischemic stroke, microglia polarize toward the classical pro-inflammatory type (M1-like) or the alternative protective type (M2-like) for a certain period of time and under different conditions, respectively, to promote intracranial inflammation, exert an anti-inflammatory and nerve-repairing effect, and repair damaged nerve functions. Microglia play a dual role in the deleterious effects of ischemic stroke, by both protecting and controlling polarization through multiple signaling pathways. With the deepening of research, research hot spots of targeted drugs for microglial polarization are increasing year by year, providing a new therapeutic strategy for the treatment of ischemic stroke. We are looking forward to more drugs that will benefit patients.

### Limitation

Although the two microglial polarization states are well studied, some researchers in the field have questioned this and even suggested discontinuing the M1/M2 classification. The idea is that the current nomenclature derived from the study of peripheral macrophages is applied to microglia, they argue that M1/M2 class macrophage activation is useless to organize our thinking about microglia, frankly said to be destructive (130). With the deepening of research, it was found that because microglia and macrophages are homologous, many markers of these two types of cells are the same (131), so research continues to use microglia/macrophages. In addition, from 14 May 2016 to 30 May 2022, a PubMed search for “M1 M2 microglia” retrieved 1,121 articles, and the number is increasing year by year. After that, in addition to using the original M1/M2-like microglia classification, some scholars proposed additional refined phenotypes (M1 microglia, M2a microglia, M2b microglia, and M2c microglia). If the M1/M2-like microglia classification had some flaws in the research at the time, then with more research, the M1-like, M2a-like, M2b-like, and M2c-like classifications would be more of the morphology and function of microglia, which would be exactly what this review reflected.

As mentioned earlier, there are many therapeutic targets for ischemic stroke in the microglial polarization process, but there are still many problems to be studied and solved. Firstly, experimental models and basic experiments of stroke are needed, and more experimental model data must be collected and organized to confirm the authenticity of relevant views. Secondly, the transition factors between M1-like and M2-like microglia and their processes require further studies. Finally, the homeostatic regulatory mechanisms of microglial polarization are discussed in this review, and the range of potential therapy targets needs to be further explored. Then, in the future, numerous studies on microglial polarization must be conducted.

### Author contributions

YM and WX were in charge of the literature search and manuscript writing. The content of this article was made by consensus of all the authors. All the listed authors contributed substantially, directly, and intellectually to the work and approved it for publication.

### Funding

This study was funded by grants from the National Natural Science Foundation of China (No. 82060225), the Guangxi Natural Science Foundation (No. 2018GXNSFAA281151 and 2020GXNSFAA297154), and the Scientific Research Project of Guangxi Health Commission (No. S2018020).

### Conflict of interest

The authors declare that the research was conducted in the absence of any commercial or financial relationships that could be construed as a potential conflict of interest.

## Publisher's note

All claims expressed in this article are solely those of the authors and do not necessarily represent those of their affiliated organizations, or those of the publisher, the editors and the reviewers. Any product that may be evaluated in this article, or claim that may be made by its manufacturer, is not guaranteed or endorsed by the publisher.

## Abbreviations

SBI: Secondary brain injury
BBB: Blood–brain barrier
CNS: Central Nervous System
INF-γ: Interferon-γ
LPS: Lipopolysaccharide
TNF- α: Tumor necrosis factor-α
IL: interleukin
Ym1 [chitinase-like protein 3 (Chil3)]
Ym1/2: chitinase-3-like protein 3
ICAM1: intercellular adhesion molecule 1
STAT: Signal transducer and activator of transcription
JAK: janus kinase
NO: nitrous oxide
SDF-1/CXCL12: Stromal cell-derived factor-1
CD: Cluster of Differentiation
MHC II: Major histocompatibility complex class II
Arg-1: Arginase-1
TGF-β: Transforming growth factor-β
IGF-1: Insulin growth factor 1
A2a R: Adenosine A2a Receptor
iNOS: Inducible nitric oxide synthase
MCAO: Middle cerebral artery occlusion
MMP-9: Matrix Metalloproteinase 9
COX-2: Cyclooxygenase-2
EGFs: Epidermal growth factors
NRR: Negative Regulatory Region
HD: Heterodimerization domain
TLRs: Toll-like receptors
MAPK: Mitogen-activated protein kinase
MMP: Metalloproteinase
PParγ: Peroxisome proliferator-activated receptor γ
PNS: peripheral nervous system
Nrf2: Nuclear Factor erythroid 2-Related Factor 2
PPAR: Peroxisome proliferator-activated receptor
DEX: Dexmedetomidine
TSPO: Translocator protein
MCAO: Middle cerebral artery occlusion
NLRP3: Nod-like receptor pyrin domain-containing protein 3
ICAM-1: Intercellular cell adhesion molecule-1
Aβ: Amyloid-β
ROS: Reactive oxygen species
RNS: Reactive nitrogen species
HTT: Huntingtin
mSOD1: Mutant human superoxide dismutase1
DHEA: Dehydroepiandrosterone
TrkA: tropomyosin-associated kinase A
Akt: protein kinase B
Jmjd3: Histone 3 Lysine 27 (H3K27) demethylase Jumonji D3
TREM2: trigger receptor 2
DHA: Docosahexaenoic acid
MANF: Mesencephalic astromarch-derived neurotrophic factor.
